# Privacy-preserving of endoscopy documentations with reasoning-aware focused large language models

**DOI:** 10.1016/j.igie.2025.05.009

**Published:** 2026-03-30

**Authors:** Partha Pratim Ray

**Affiliations:** Department of Computer Applications, Sikkim University, Gangtok, Sikkim, India

*Editor-in-Chief comment on letter:*

*Artificial intelligence (AI) is rapidly encroaching on many aspects of our lives, including in health care and in scientific writing. Its possible future impact on the broader workflow of physicians and scientists appears far-reaching. The following letter highlights the potential of sophisticated large language models to analyze and create content, and it makes this specific point by using generative AI to editorialize the referenced paper. While AI may become accepted for creating content in the future, its current use in writing articles remains appropriately limited to improving readability and language, with this letter representing a unique exception to illustrate the capability of current large language models.*

**Linda S. Lee, MD, FACG, FASGE**

*iGIE* Editor-in-Chief

To the editor,

The recent publication “Deep sight: enhancing periprocedural adverse event recording in endoscopy by structuring text documentation with privacy-preserving large language models” by Wiest et al^1^ exploring privacy-preserving large language models (LLMs) for structuring endoscopy reports, presents significant advancements in extracting adverse event details efficiently and accurately. The demonstrated effectiveness of generative pre-trained transformer (GPT)-4 (OpenAI, San Francisco, Calif, USA) and locally deployable Llama 2 (Meta, Menlo Park, Calif) variants in capturing structured clinical information from free-text documentation represents a meaningful step toward scalable and precise clinical documentation practices.

Nevertheless, widespread clinical adoption of proprietary cloud-based models such as GPT-4 inherently raises important privacy concerns, particularly when dealing with sensitive patient data.^2^^,^^3^ Alternatively, fine-tuned open-source models, such as the German-language-specific Llama 2 or Llama 3 variant “SauerkrautLM,” provide high sensitivity and specificity with the added benefit of local deployment capabilities, thereby significantly enhancing patient data security and privacy.

Future investigations should consider incorporating even more advanced reasoning-capable models such as O3 (xAI, Memphis, Tenn, USA), DeepSeek-R1 (DeepSeek, Hangzhou, China), Grok 3 (xAI, Memphis, Tenn), Claude 3.7 (Anthropic AI, San Francisco, Calif), and QwQ-32B (Alibaba Cloud, Hangzhou, China). These models, characterized by higher billion-parameter counts (eg, 32B or more) and sophisticated reasoning abilities, hold promise for significantly improving the accurate interpretation of complex medical scenarios and reducing misclassifications in clinical documentation tasks.

Critically, addressing privacy comprehensively involves both model selection and deployment strategies. Leveraging advanced LLMs within decentralized, localized edge-computing frameworks can ensure the data remain securely on-site, effectively mitigating privacy risks.^4^ A privacy-centric implementation could substantially increase clinician acceptance and streamline workflows without compromising patient confidentiality.^5^

As a final remark, the presented article underscores the potential of fine-tuned LLMs in structuring clinical documentation while highlighting the importance of adopting advanced reasoning models within robust, privacy-aware frameworks. Such integration is pivotal for secure, accurate, and clinically practical applications.

## Declaration of generative AI and AI-assisted technologies in the writing process

During the preparation of this work, the author used GPT-4.5 in order to provide in-depth analysis and the initial draft of the article. After using this tool/service, the author reviewed and edited the content as needed and takes full responsibility for the content of the publication.

## Disclosure

The author declares that they have no known competing financial interests or personal relationships. They declare that no funding has been received for this work/letter/correspondence.
